# Clinical implications of c-maf expression in plasma cells from patients with multiple myeloma

**DOI:** 10.1186/s40164-017-0076-3

**Published:** 2017-05-26

**Authors:** GuoQing Wei, LiJun Wang, HanJin Yang, XiaoYan Han, GaoFeng Zheng, WeiYan Zheng, Jie Sun, JiMin Shi, WenJun Wu, Yi Zhao, DongHua He, Bo Wang, Zhen Cai, JingSong He

**Affiliations:** 1 Department of Hematology, Bone Marrow Transplantation Center, The First Affiliated Hospital, College of Medicine, Zhejiang University, Hangzhou, China; 2 Department of Pathology, The First Affiliated Hospital, College of Medicine, Zhejiang University, Hangzhou, China

**Keywords:** Multiple myeloma, c-maf, Immunohistochemistry, Therapy response, Prognosis

## Abstract

**Background:**

Multiple myeloma (MM) is a type of hematological malignancy with significant heterogeneity in clinical features and prognosis. Cytogenetic abnormalities are the major factors affecting patient outcomes. Studies have shown that immunohistochemistry (IHC)-based detection of cancer-related genes expression could be alternative indicators for the prognosis of MM.

**Methods:**

Nuclear expression of c-maf protein in the bone marrow plasma cells of 128 multiple myeloma patients were examined by IHC, and its association with the clinicopathological features of MM patients was analyzed as well.

**Results:**

Among the 128 patients, the positive rate of c-maf protein expression was up to 30.5%, which had no correlation with patient age, M protein type, Durie-Salmon staging system, the International Staging System, abnormal plasma cell ratio in the bone marrow, or the level of peripheral blood hemoglobin, serum calcium or lactate dehydrogenase. However, the c-maf-positive patients had a significantly higher rate of hypoproteinemia (*p* = 0.026) and higher serum β2-microglobulin levels (>2500 μg/L) (*p* = 0.007). Patients with negative c-maf expression had higher remission rates upon the treatment of non-bortezomib-based regimens although no effect of c-maf expression on progression-free survival or overall survival was observed.

**Conclusion:**

Patients with negative c-maf expression had higher remission rates upon the treatment of non-bortezomib-based regimens although no effect of c-maf expression on survival was observed. A further large-scale prospective study is required to verify these findings.

## Background

Multiple myeloma (MM) is a type of monoclonal plasma cell dysplasia among hematological malignancies, often leading to renal insufficiency, bone damage, anemia, and other terminal visceral damage. MM often has a relatively long period of benign pre-disease state called ‘monoclonal gammopathy of undetermined significance’ (MGUS). Approximately 1% of patients with MGUS progress to MM every year [1, 2]. There is significant heterogeneity in the clinical features and prognosis of MM patients. Diagnostic clinical and biochemical staging systems are commonly used in the evaluation of MM. However, MM patients at the same stage may still have different disease progression [2, 3]. Cytogenetic abnormalities have become increasingly important in prognostic risk stratification and management of MM [4–7]. Conventional karyotype analysis and fluorescence in situ hybridization (FISH) are common methods for the analysis of cytogenetic abnormalities in MM. Compared to conventional karyotype analysis, which has a real lower detection rate of abnormal karyotypes, the higher detection rate of FISH make it to be a primary approach in cytogenetic analysis [4, 8].

However, there are many cell surface molecules and intracellular molecular markers that abnormally expressed in malignant plasma cells, which are closely related to disease prognosis [9–13]. Studies have shown that the use of immunohistochemistry (IHC) to detect the expression of cancer-related genes, such as fibroblast growth factor receptor 3 (FGFR3), the tumor suppressor gene p53, and transcription factor c-maf can be an alternative method for the prognostic analysis of MM [14–17]. In this study, we utilized immunohistochemistry to examine the expression of the transcription factor c-maf, the cellular homolog of v-maf, in MM patients in China to retrospectively analyze its correlation with disease characteristics, treatment efficacy, and patient survival.

## Methods

### Patients

This study began on November 15, 2016, and a total of 128 MM patients who were treated in our hospital from January 1, 2009 to May 31, 2012, including 112 newly diagnosed and 16 recurrent cases, were recruited. There were 84 males and 44 females with a median age of 60 years (ranging from 31 to 88 years). In terms of the types of M protein heavy chains, 31 cases were IgA, 6 cases were IgD, 55 cases were IgG, and 36 cases were light chain. For light chain types, 60 cases were κ-light chain and 68 cases were λ-light chain. With respect to the Durie-Salmon staging system, four cases were 1A, 10 cases were 2A, 85 cases were 3A, and 29 cases were 3B. According to the International Staging System (ISS), 17 cases were stage 1, 55 cases were stage 2, and 56 cases were stage 3.

### Treatment regimens

Among the 128 patients, 116 received systemic treatment and were included in the statistical analysis of therapeutic efficacies of MM treatments. Of these 116 patients, 70 received bortezomib-based combination chemotherapies, mainly including a two-drug combination regimen with bortezomib and dexamethasone (PD) or three drug combination regimens with PD and cyclophosphamide (PCD), PD and doxorubicin (PAD), and PD and thalidomide (PTD) (see our previous study [18] for the specific treatment plans). The other 46 patients received thalidomide-based regimen or traditional chemotherapy, including thalidomide together with dexamethasone and cyclophosphamide or doxorubicin, or a VAD regimen only (vindesine together with doxorubicin and dexamethasone), with a median of three courses of treatment (1–8 courses). Seven patients received autologous hematopoietic stem cell transplantation, including five cases with bortezomib treatment and two cases with non-bortezomib-based treatment.

### Immunohistochemical analysis

Anti-CD138 antibody (1:100, Serotec, Oxford, UK) was used to label the plasma cells, and anti-c-maf monoclonal antibody (Vector Laboratories, Burlingame, CA) was used for immunohistochemical analysis in this study, followed by labeling with biotin-streptavidin-horse peroxidase and NovaRed substrate development.

Bone marrow biopsies were collected from all 128 MM patients for routine diagnosis or assessment of disease condition by conventional decalcification, fixation, paraffin-embedding, and 5-µm sample sectioning. After deparaffinization and heat-induced antigen retrieval of the bone marrow biopsies, anti-CD138 monoclonal antibody was used to detect the plasma cells in the bone marrow. c-maf protein expression in >20% of plasma cells in the bone marrow was defined as positive expression. Paraffin-embedded human skin biopsies were used as the positive control for c-maf immunostaining. Non-immune mouse serum was used to replace the primary antibody as a negative control. Immunohistochemical analysis of c-maf expression was performed separately by two pathologists. For any discrepancy in diagnosis, a third pathologist was assigned to adjudicate the final diagnosis.

### Response criteria

In this study, the responses to different treatments were classified into five categories: complete remission, partial remission (PR), very good partial response (VGPR), stable disease (SD), and progressive disease (PD). The specific evaluation criteria were in accordance with the consensus criteria of therapeutic efficacies set forth by the International Myeloma Working Group (IMWG), 2006 [19]. The therapeutic efficacies of different treatments were evaluated every 30–60 days during the treatment period.

### Statistical analysis

SPSS 18.0 (SPSS Inc., Chicago, IL) software was used for statistical analysis in this study. Overall survival (OS) is defined as the time from therapy initiation to death from any cause or loss of follow-up. Progression-free survival (PFS) refers to the time elapsed between therapy initiation and disease progression or death from any cause. Pearson’s Chi square test or Fisher’s exact test were used for the comparison of frequency distribution between different groups. Independent samples *t* test was used to compare the difference of mean values between two groups. Kaplan–Meier method was used for the analysis of recurrence-free survival and OS of the patients. Statistical significance was defined at *p* < 0.05.

## Results

### Positive expression rate of c-maf protein

**Fig. 1:**
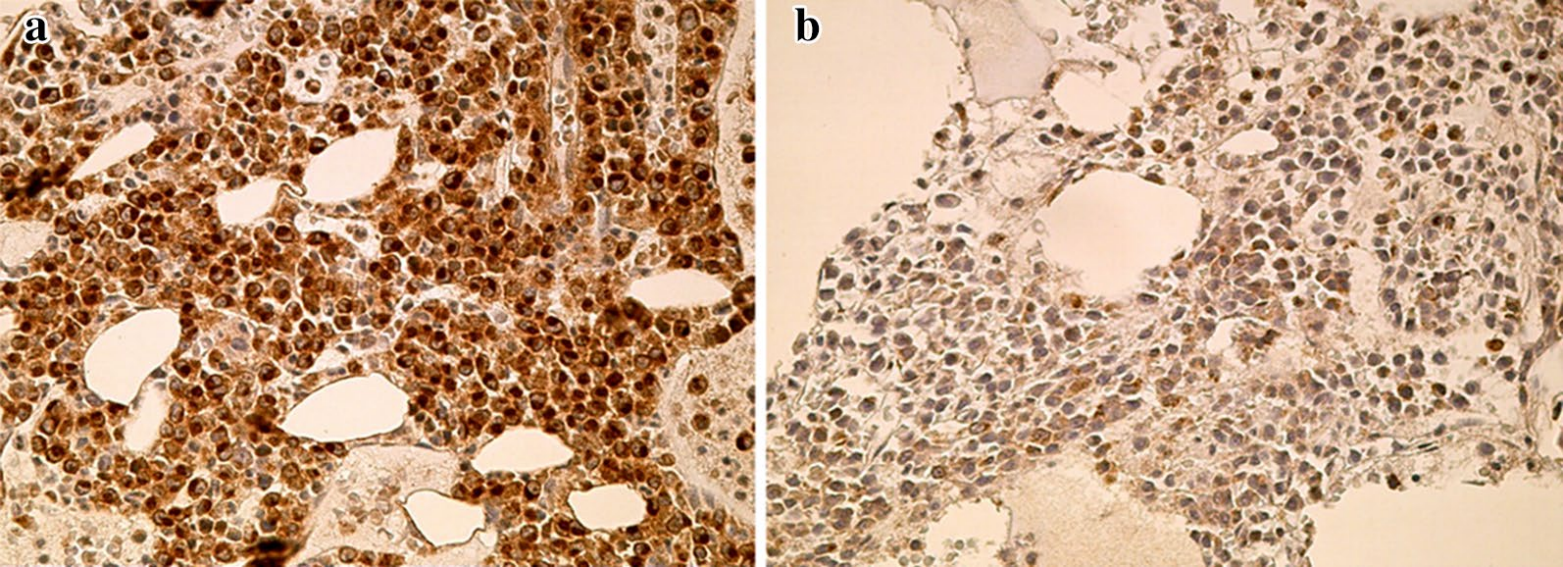
c-maf immunoreactivity in multiple myeloma. **a** Positive reaction with a bone marrow biopsy from a myeloma patient. **b** Negative reaction with a bone marrow biopsy from a myeloma patient

Among the 128 MM patients, 39 patients (30.5%) displayed c-maf expression in >20% plasma cells in the bone marrow and were thus categorized as c-maf-positive patients (Fig. 1).

### Correlation between c-maf expression and disease characteristics of MM patients

The positive expression of c-maf was not significantly correlated with patient gender, age, M protein type, Durie-Salmon staging system, the ISS, abnormal plasma cell ratio in bone marrow, peripheral blood hemoglobin, serum calcium and lactate dehydrogenase levels, and disease stage (incidence or recurrence, *p* > 0.05, Table 1). However, the c-maf-positive MM patients had a higher tendency of hypoalbuminemia (χ^2^ = 4.971, *p* = 0.026) than the c-maf-negative MM patients. The serum albumin levels of 25 out of 39 c-maf-positive MM patients were lower than the normal level (35 g/L, 64.1%), while only 42.7% (n = 38 out of 89) c-maf-negative patients had hypoalbuminemia. In addition, our results showed that in MM patients, the c-maf expression in the bone marrow plasma cells was significantly correlated with the serum β2-microglobulin levels in the peripheral blood. Only two (8.0%) out of 25 MM patients with normal serum β2-microglobulin levels in the peripheral blood had positive expression of c-maf, while 37 (35.9%) out of 103 MM patients with significantly high serum β2-microglobulin levels had positive expression of c-maf (χ^2^ = 7.403, *p* = 0.007).

**Table 1:** Correlation between c-maf expression and disease characteristics of multiple myeloma patients

Characteristics	All patients (n = 128)	C-maf-positive (n = 39)	C-maf-negative (n = 89)	*p* value
Sex, F/M	44/84	11/28	33/56	0.331
Age, years, n (%)				
≤60	66 (51.6)	20 (51.3)	46 (51.7)	
>60	62 (48.4)	19 (48.7)	43 (48.3)	0.966
Isotype, IgH, n (%)				
IgA	31 (24.2)	9 (23.1)	22 (24.7)	
IgD	6 (4.7)	1 (2.6)	5 (5.6)	
IgG	55 (43.0)	21 (53.8)	34 (38.2)	
Light chains	36 (28.1)	8 (20.5)	28 (31.5)	0.342
Isotype, IgL, n (%)				
κ	60 (46.9)	17 (43.6)	43 (48.3)	
λ	68 (53.1)	22 (56.4)	46 (51.7)	0.622
D–S stage, n (%)				
1A + 2A	14 (10.9)	2 (5.1)	12 (13.5)	
3A	85 (66.4)	30 (76.9)	55 (61.8)	
3B	29 (22.7)	7 (17.9)	22 (24.7)	0.176
ISS stage, n (%)				
I + II	72 (56.3)	20 (51.3)	52 (58.4)	
III	56 (43.8)	19 (48.7)	37 (41.6)	0.453
Alb level, n (%)				
≥35 g/L	65 (50.8)	14 (35.9)	51 (57.3)	
<35 g/L	63 (49.2)	25 (64.1)	38 (42.7)	0.026
β2-MG level, n (%)				
≤2500 μg/L	25 (19.5)	2 (5.1)	23 (25.8)	
>2500 μg/L	103 (80.5)	37 (94.9)	66 (74.2)	0.007
Hb, n (%)				
<80 g/L	63 (49.2)	21 (53.8)	42 (47.2)	
≥80 g/L	65 (50.8)	18 (46.2)	47 (52.8)	0.488
Plt, n (%)				
≤140 × 10^9^/L	62 (48.4)	20 (51.3)	42 (47.2)	
>140 × 10^9^/L	66 (51.6)	19 (48.7)	47 (52.8)	0.670
Calcium level, n (%)				
≤2.54 mmol/L	110 (85.9)	35 (89.7)	75 (84.3)	
>2.54 mmol/L	18 (14.1)	4 (10.2)	14 (15.7)	0.412
CRP level, n (%)				
≤8.0 mg/L	92 (71.9)	29 (74.4)	63 (70.8)	
>8.0 mg/L	36 (28.1)	10 (25.6)	26 (29.2)	0.679
LDH level, n (%)				
≤250 U/L	105 (82.0)	31 (79.5)	74 (79.8)	
>250 U/L	23 (18.0)	8 (20.5)	15 (20.2)	0.620

*ISS* international stage system, *Alb* albumin, *β2-MG* β2-microglobulin, *CRP* c-reactive protein, *LDH* lactate dehydrogenase

### Correlation between c-maf expression and treatment responses of MM patients

No significant differences were found between MM patients with negative and positive expression of c-maf who received either bortezomib- or non-bortezomib-based regimen and the number of courses of therapy (*p* > 0.05). In addition, no significant differences were found between the overall response rate (ORR) to treatment (PR and above) and VGPR or better response to treatment. Our analysis of MM patients who received three and more courses of therapy showed that there was no significant difference between ORR and c-maf expression. However, the c-maf-negative MM patients displayed higher VGPR and better treatment response rates albeit statistically insignificant (77.8% vs. 58.3%, *p* = 0.090). Further analysis in the MM patients who received bortezomib demonstrated similar therapeutic efficacy in patients with negative and positive expression of c-maf, with 78.1 and 81.2% of MM patients achieving VGPR and better therapeutic efficacy, respectively (*p* = 0.800). However, among the MM patients who received non-bortezomib-based treatment, the c-maf-negative MM patients had significantly better therapeutic efficacy than the c-maf-positive MM patients, with 76.9 and 12.5% of MM patients achieving VGPR and better therapeutic efficacy, respectively (*p* = 0.003, Table 2).

**Table 2:** Correlation between c-maf expression and treatment response of multiple myeloma patients

	All patients (n = 116)	C-maf-positive (n = 36)	C-maf-negative (n = 80)	p value
Therapy, n (%)				
Bor	70 (60.3)	22 (61.1)	48 (60.0)	
Non-bor	46 (39.7)	14 (38.9)	32 (40.0)	0.910
Courses, n (%)				
<3	44 (37.9)	11 (30.6)	33 (41.3)	
≥3	72 (62.1)	25 (69.4)	47 (58.7)	0.272
Response (ORR), n (%)				
<PR	50 (46.9)	12 (43.6)	38 (48.3)	
≥PR	66 (53.1)	24 (56.4)	42 (51.7)	0.154
Response (VGPR), n (%)				
<VGPR	63 (54.3)	22 (61.1)	41 (51.3)	
≥VGPR	53 (45.7)	14 (38.9)	39 (48.7)	0.324

Courses ≥3	N = 69	N = 24	N = 45	
Response (ORR), n (%)				
<PR	7 (10.1)	3 (12.5)	4 (8.9)	
≥PR	62 (89.9)	21 (87.5)	41 (91.1)	0.641
Response (VGPR), n (%)				
<VGPR	20 (29.0)	10 (41.7)	10 (22.2)	
≥VGPR	49 (71.0)	14 (58.3)	35 (77.8)	0.090

Bortezomib	N = 48	N = 16	N = 32	
Response (ORR), n (%)				
<PR	4 (8.3)	1 (6.3)	3 (9.4)	
≥PR	44 (91.7)	15 (93.7)	29 (90.6)	0.712
Response (VGPR), n (%)				
<VGPR	10 (20.8)	3 (18.8)	7 (21.9)	
≥VGPR	38 (79.2)	13 (81.2)	25 (78.1)	0.800

Non-bortezomib	N = 21	N = 8	N = 13	
Response (ORR), n (%)				
<PR	3 (14.3)	2 (25.0)	1 (7.7)	
≥PR	18 (85.7)	6 (75.0)	12 (92.3)	0.278
Response (ORR), n (%)				
<VGPR	10 (47.6)	7 (87.5)	3 (23.1)	
≥VGPR	11 (52.4)	1 (12.5)	10 (76.9)	0.003

*Bor* bortezomib, *ORR* overall response rate, *VGPR* very good partial response

### Correlation between c-maf expression and survival of MM patients

**Fig. 2:**
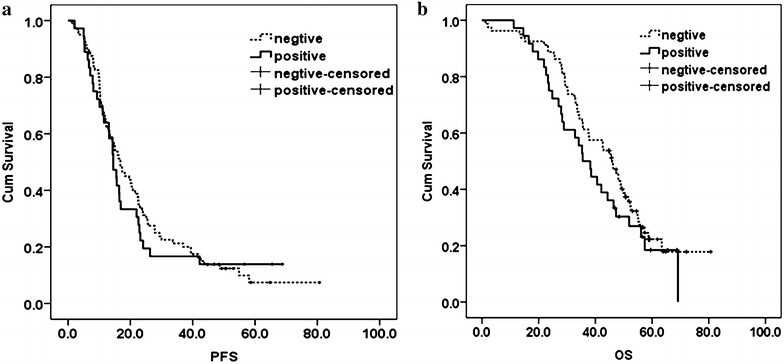
Survival of MM patients. Progression-free survival (**a**) and overall survival (**b**) of multiple myeloma patients according to c-maf expression by IHC—36 patients with positive expression and 80 patients with negative expression of c-maf

All MM patients who received treatment at our hospital (n = 116) were followed up until December 31, 2015, with a median duration of follow-up of 42.8 months. Among them, 102 MM patients (87.9%) exhibited disease progression, including 71 c-maf-negative MM patients (88.7% of all c-maf-negative MM patients) and 31 c-maf-positive MM patients (86.1% of all c-maf-positive MM patients) who had a median PFS of 15.6 months (95% CI 13.6–17.6 months). Whereas the median PFS of the c-maf-negative MM patients was 16.8 months (95% CI 13.3–20.3), that of the c-maf-positive MM patients was 14.5 months (95% CI 11.8–17.2); and no significant difference was found between the two groups of patients (*p* = 0.658). The 3-year PFS of the c-maf-negative and c-maf-positive MM patients were 20.0 and 16.7%, respectively (Fig. 2a).

Eighty MM patients died at the end of the follow-up period, including 74 cases from disease progression, four cases from septic shock, one case from sudden cardiac death and one case from other causes of death. Among the patients who dies, 53 were c-maf-negative MM patients (66.2%) and 27 were c-maf-positive MM patients (75.0%), with a median OS of 42.6 months among the patients (95% CI 35.3–49.9 months). The median OS of c-maf-negative MM patients was 45.9 months (95% CI 40.9–50.9 months), while that of c-maf-positive MM patients was 35.5 months (95% CI 29.0–42.0 months), with no significant difference between the two groups (*p* = 0.233). The 3-year OS of c-maf-negative and c-maf-positive MM patients were 61.3 and 50.0%, respectively (Fig. 2b).

## Discussion

Cytogenetic changes are important factors involved in the incidence and development of MM and are closely associated with drug resistance. Furthermore, cytogenetic changes affect the prognosis of MM and thus are considered as an important tool for the decision-making process in clinical treatment. Chromosomal translocations in the immunoglobulin heavy chain (IgH) locus are the common chromosomal abnormalities in MM [2, 4, 6], with the most common types including t(4;14), t(11;14), and t(14;16). These result in the structural hyperactivation of fibroblast growth factor receptor 3 (FGFR3), cyclin D1, and the transcription factor c-maf, thereby regulating the proliferation, apoptosis, and biological behaviors of MM cells [5, 14, 15].

The presence of t(14;16) chromosomal abnormality in MM patients has been generally considered an indicator of poor prognosis [4–7, 20, 21]. A study showed that MM patients with t(14;16) have a median survival of only 16 months, while those without t(14;16) have a median survival of 41 months [22]. Most of clinical guidelines for the diagnosis and treatment of myeloma consider t(14;16) as one of the major criteria of high-risk patients [2, 5–7]. However, some researchers have offered alternative explanations and do not consider the prognostic significance of t(14;16) [23, 24]. They suggested that the poor prognosis of MM might actually be associated with the presence of +1q21 and del(17p13) [24]. In addition, the incidence of t(14;16) in MM is quite low and only present in approximately 1.0–4.6% of advanced MM patients [20, 22–25]. Data from recent studies in China also showed that the incidence of t(14;16) was only 0.8–6.7% among newly diagnosed MM patients [26–28], and the detection rate of t(14;16) was 1.2% in recently diagnosed MM patients [26]. A recent study from Hong Kong showed the absence of t(14;16) among 40 MM cases [29]. Translation of t(14;16) has also been observed in patients with MGUS, with the detection rate of approximately 5% [1, 30], suggesting that the chromosomal translation may be involved in the early stages of the disease; however, its impacts on disease prognosis remains controversial.

In this study, we used IHC to detect c-maf protein expression in the bone marrow plasma cells of MM patients and evaluated the impact of c-maf protein expression on the pathology and prognosis of MM. Among the 128 MM patients, 39 (30.5%) displayed high c-maf expression, which was concordant with the findings of previous studies [17, 31]. Chang et al. [14–17] studied the expression of p53, FGFR3, and c-maf protein in MM cells and found that the incidence of high c-maf protein expression in MM cells was significantly higher than the t(14;16) chromosomal abnormality. In addition, among the 73 MM patients examined by Chang et al., 22 expressed c-maf, while only 4 patients displayed the t(14;16) chromosomal translocation as detected by FISH [17]. Hurt et al. [31] used RT-PCR to detect the expression of *c*-*maf* mRNA in MM cell lines and plasma cells in the bone marrow of MM patients and showed that 46% (13/28) of the cell lines had high c-maf expression, whereas only 6 of the cell lines harbored the t(14;16) abnormality. In addition, up to 50% of MM patients (13/26) had high c-maf expression in the bone marrow cells, suggesting that 16q23 and 14q32 translocations were not the only cause of high c-maf expression. All these results demonstrated that the expression of c-maf is significantly high in MM patients, highlighting the importance of immunohistochemistry-based examination of c-maf expression. Of note, the correlations between *c*-*maf* mRNA and c-maf protein expression and t(14;16) currently still remain controversial [14, 17, 31–33]. Rasmussen et al. [32] and Natkunam et al. [33] showed that approximately 5% of the bone marrow plasma cells of MM patients expressed c-maf mRNA and protein, with a similar detection rate as that of t(14;16) and significant correlation with t(14;16). However, it is unclear whether the inconsistent findings are due to differences in the evaluation of IHC and FISH.

The t(14;16) chromosomal abnormality of myeloma cells indicates poor prognosis of MM. However, the relationship between *c*-*maf* gene expression and MM characteristics and prognosis had not been extensively explored. Our results suggested that the expression of c-maf protein was associated with serum albumin and β2-microglobulin levels in MM patients. In addition, the expression of c-maf protein in MM cells did not have any significant correlation with treatment response, PFS, and OS of MM patients. Nevertheless, the sub-group analysis demonstrated that patients who received non-bortezomib-based and thalidomide-based regimens or traditional chemotherapy displayed less VGPR or better treatment response if they had high levels of c-maf expression that if they were negative for c-maf expression, suggesting that c-maf-positive patients exhibited some degree of drug resistance, which could be overcome with bortezomib.

Therefore, further studies are necessary to identify the relationship between the expression of the transcription factor c-maf and the t(14;16) chromosomal translocation, as well as the relationship between the abnormal expression of *c*-*maf* gene and disease characteristics and prognosis of MM to clarify the significance of c-maf in the course of MM. Nevertheless, studies have shown that c-maf may be involved in the regulation of the cell cycle and promotion of growth, survival and angiogenesis of MM cells and facilitation of interactions between MM cells and tumor microenvironment [31, 34]. Thus, c-maf may be a potential therapeutic target for MM.

## Conclusion

In this study, it was shown that the rate of expression of the transcription factor c-maf in MM patients was higher than the incidence of translocation of chromosomes 14 and 16 as reported earlier. The positive expression of c-maf may be correlated with hypoproteinemia and elevated serum β2-microglobulin levels in MM patients. Moreover, the c-maf-negative patients had better remission, especially with non-bortezomib-based treatments although c-maf expression displayed no significant correlation with the prognosis of MM patients.

## Abbreviations

MM: multiple myeloma
IHC: immunohistochemistry
ISS: International Staging System
PFS: progression-free survival
OS: overall survival
MGUS: monoclonal gammopathy of undetermined significance
FGFR3: fibroblast growth factor receptor 3
PR: partial remission
VGPR: very good partial response
SD: stable disease
PD: progressive disease
IMWG: International Myeloma Working Group
ORR: overall response rate
